# Phenotypic and Genomic Analysis of *Clostridium beijerinckii* NRRL B-598 Mutants With Increased Butanol Tolerance

**DOI:** 10.3389/fbioe.2020.598392

**Published:** 2020-11-05

**Authors:** Maryna Vasylkivska, Barbora Branska, Karel Sedlar, Katerina Jureckova, Ivo Provaznik, Petra Patakova

**Affiliations:** ^1^Department of Biotechnology, University of Chemistry and Technology, Prague, Prague, Czechia; ^2^Department of Biomedical Engineering, Faculty of Electrical Engineering and Communication, Brno University of Technology, Brno, Czechia

**Keywords:** butanol tolerance, random chemical mutagenesis, solventogenic *Clostridium* species, genome sequence, butanol efflux

## Abstract

*N*-Butanol, a valuable solvent and potential fuel extender, can be produced via acetone-butanol-ethanol (ABE) fermentation. One of the main drawbacks of ABE fermentation is the high toxicity of butanol to producing cells, leading to cell membrane disruption, low culture viability and, consequently, low produced concentrations of butanol. The goal of this study was to obtain mutant strains of *Clostridium beijerinckii* NRRL B-598 with improved butanol tolerance using random chemical mutagenesis, describe changes in their phenotypes compared to the wild-type strain and reveal changes in the genome that explain improved tolerance or other phenotypic changes. Nine mutant strains with stable improved features were obtained by three different approaches and, for two of them, ethidium bromide (EB), a known substrate of efflux pumps, was used for either selection or as a mutagenic agent. It is the first utilization of this approach for the development of butanol-tolerant mutants of solventogenic clostridia, for which generally there is a lack of knowledge about butanol efflux or efflux mechanisms and their regulation. Mutant strains exhibited increase in butanol tolerance from 36% up to 127% and the greatest improvement was achieved for the strains for which EB was used as a mutagenic agent. Additionally, increased tolerance to other substrates of efflux pumps, EB and ethanol, was observed in all mutants and higher antibiotic tolerance in some of the strains. The complete genomes of mutant strains were sequenced and revealed that improved butanol tolerance can be attributed to mutations in genes encoding typical stress responses (chemotaxis, autolysis or changes in cell membrane structure), but, also, to mutations in genes X276_07980 and X276_24400, encoding efflux pump regulators. The latter observation confirms the importance of efflux in butanol stress response of the strain and offers new targets for rational strain engineering.

## Introduction

Butanol can be produced from renewable feedstocks of different kinds, including agricultural waste materials, by acetone-butanol-ethanol (ABE) fermentation using solventogenic clostridia (Lee et al., 2008). The most famous species of the group of solventogenic clostridia are *Clostridium acetobutylicum* and *Clostridium beijerinckii*, both of which share a common process bottleneck – low tolerance to butanol.

Butanol is a toxic metabolite that tends to incorporate into the cell membrane, increases membrane fluidity and may disrupt membrane functions (Bowles and Ellefson, 1985; Lepage et al., 1987; Patakova et al., 2018; Vasylkivska and Patakova, 2020). In *C. acetobutylicum*, at inhibitory levels, effects of butanol on cell membrane results in lower ATP generation by the cell, a malfunction in nutrient uptake and an inability of the cell to maintain its internal pH (Bowles and Ellefson, 1985). In Gram-negative bacteria, butanol damages the inner and outer membranes and can also result in a change in cell shape (Fletcher et al., 2016). Such damage, at the cellular level, results in low culture viability and decreased growth rate and, as a consequence, low final butanol concentrations achieved after fermentation (Ingram, 1986).

Different methods have been used to obtain butanol-tolerant strains of solventogenic clostridia, including targeted modifications, e.g., overexpression of genes encoding heat-shock proteins or modification of fatty acids synthesis (Tomas et al., 2003; Zhao et al., 2003; Mann et al., 2012; Liao et al., 2017), serial transfer and adaptation (Lin and Blaschek, 1983; Baer et al., 1987; Soucaille et al., 1987; Xue et al., 2012; Liu et al., 2013; Yang and Zhao, 2013) or random mutagenesis (Hermann et al., 1985; Matta-el-Ammouri et al., 1986; Annous and Blaschek, 1991; Jain et al., 1994; Mao et al., 2010; Kong et al., 2016; Tanaka et al., 2017). Use of these methods resulted in improved survival in the presence of butanol at a concentration of 10–12 g/L for the wild-type strain (WTS) to 16–18 g/L for mutant strains (Vasylkivska and Patakova, 2020), sometimes up to 23 g/L, as in the case of mutant strain *C. beijerinckii* BA101 (Qureshi and Blaschek, 2001). Surprisingly, random and targeted mutagenesis have apparently produced very similar improvements in butanol tolerance, although this probably reflects our incomplete understanding of butanol tolerance mechanisms and their regulation (Patakova et al., 2018). In most cases, obtained mutant strains in addition to higher tolerance exhibited higher butanol production, usually an improvement from approximately 9–12 g/L for WTS to 13–16 g/L for mutant strains, sometimes up to 19–21 g/L for selected mutants (Vasylkivska and Patakova, 2020). However, some authors have reported increased tolerance, but not increased butanol production for obtained mutants, both for random (Baer et al., 1987; Gallardo et al., 2017; Máté de Gérando et al., 2018) and targeted (Zhao et al., 2003; Alsaker et al., 2004; Mann et al., 2012; Jones et al., 2016) mutagenesis. Thus, although it is commonly accepted that increased butanol tolerance leads to higher production, such data suggest strain-specific dependence between butanol tolerance and production or even the absence of any direct connection.

Efflux is one of the innate mechanisms of stress response in bacteria and it is based on active transport of the substances from the cell. This mechanism is mostly associated with antibiotic resistance, however, it has been shown that it also takes part in the butanol stress response of *Pseudomonas putida* and *Escherichia coli* (Fisher et al., 2014; Bui et al., 2015; Basler et al., 2018; Zhang et al., 2018). It was suggested that efflux can be very effective when cells are dealing with high solvent concentrations (Segura et al., 2012), and enhancement of efflux pump activity could possibly shift metabolic flux, resulting in higher production (Mukhopadhyay, 2015).

Efflux, particularly butanol efflux, is rarely studied in solventogenic clostridia (Vasylkivska and Patakova, 2020). Up-regulation of ATP-binding cassette transporters (Schwarz et al., 2012) and putative efflux pump regulators (Sedlar et al., 2019) observed under cultivation with butanol stress are the only evidence of innate butanol efflux in the group. Recently, a butanol efflux pump from *P. putida* S12 was expressed in *Clostridium saccharoperbutylacetonicum*, resulting in improved butanol tolerance (Jiménez-Bonilla et al., 2020) and demonstrating that butanol efflux studies in solventogenic *Clostridium* have potential for the development of butanol-tolerant production strains. As no native butanol efflux pumps have yet been reported for solventogenic clostridia, targeted engineering cannot be used for the study. Nevertheless, it was shown that efflux enhancement can be achieved even by a point mutation in the efflux pump gene sequence (Bohnert et al., 2007) or in the efflux pump promoter or regulator. To generate such mutations, ethyl methanesulfonate (EMS) or ethidium bromide (EB) can be used. EMS can alkylate guanine bases in DNA, resulting in unidirectional random transition mutations between GC and AT base pairs. Such a type of mutation can lead to an amino acid change and even loss of protein function. EMS was previously successfully used by Jain et al. (1994) to obtain a high butanol producing stable asporogenic mutant, *C. beijerinckii* ATCC 55025, formerly *C. acetobutylicum* ATCC 55025 (Jain et al., 1994). EB is a known substrate of different efflux pumps in both Gram-positive (Patel et al., 2010) and Gram-negative bacteria (Paixao et al., 2009) and also a chemical mutagen (Ohta et al., 2001). Use of EB may enhance efflux in the strain, resulting in improved butanol tolerance. Therefore, for this study, we have chosen three approaches to develop butanol-tolerant mutants of solventogenic *C. beijerinckii* NRRL B-598:

(1) Random chemical mutagenesis using EMS with selection on butanol (EMS + butanol mutants).
(2) Random chemical mutagenesis using EMS with selection on EB (EMS + EB mutants).
(3) Random chemical mutagenesis using EB as a mutagenic agent, where strains were selected directly on agar plates containing EB (EB mutants).

Phenotypic behavior of selected mutant strains was observed by testing tolerance to different substances such as butanol, EB, ethanol and antibiotics, and metabolite production. The complete genomes of mutant strains that exhibited improved butanol tolerance were sequenced to understand their changes in phenotype and to contribute to knowledge about mutations that lead to increased butanol tolerance. To the best of our knowledge, the genomic sequence of only a few butanol-tolerant strains obtained by random mutagenesis are available.

## Materials and Methods

### Bacterial Strain

*Clostridium beijerinckii* NRRL B-598 (WTS), former *Clostridium pasteurianum* NRRL B-598 (Sedlar et al., 2017), obtained from the Agricultural Research Service Culture Collection (1815 N. University Street, Peoria, IL 61604) was used in this study. The culture was maintained in the form of a spore suspension [containing about 2.2⋅10^8^ spores/ml, determined by method described by Branska et al. (2018)] in sterile distilled water at 4°C. For each cultivation experiment, 450 μl of spore preserve was used for 100 ml of cultivation medium.

### Mutagenesis and Strain Selection

#### Mutagenesis

**FIGURE 1:**
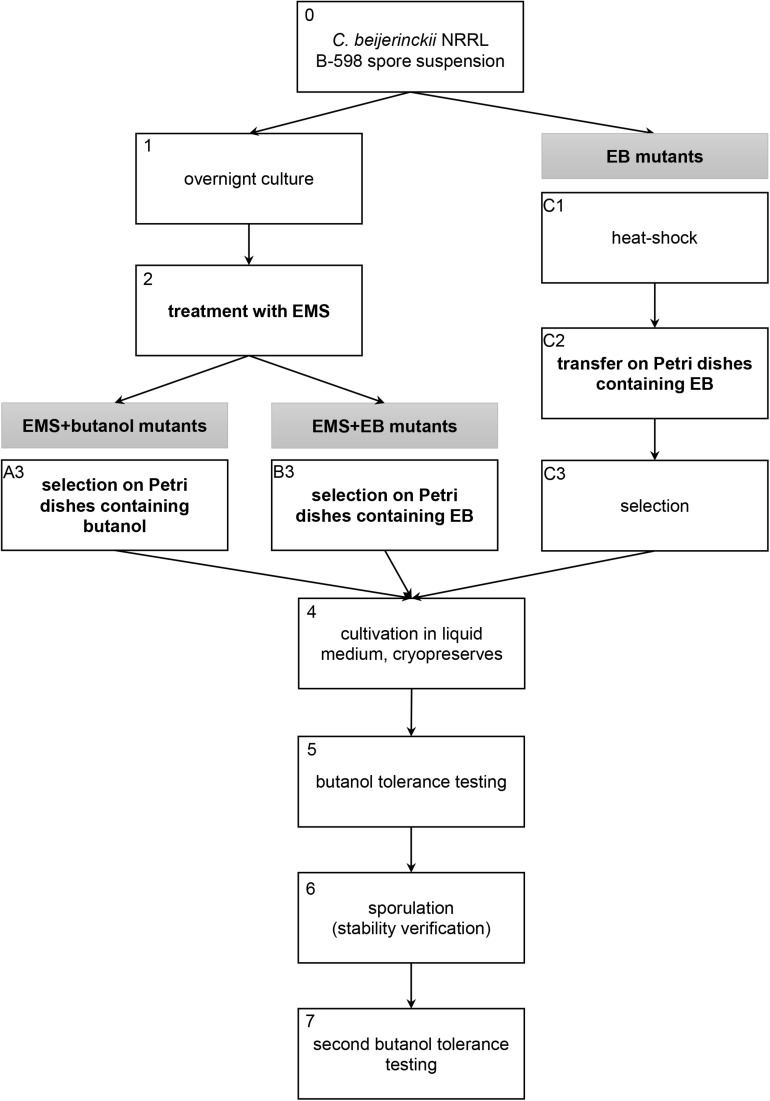
Scheme of three random chemical mutagenesis approaches that were used to obtain mutant strains of *C. beijerinckii* NRRL B-598 with increased butanol tolerance. EMS, ethyl methanesulfonate; EB, ethidium bromide.

Mutant strains described in this work were obtained using three different approaches of random chemical mutagenesis (Figure 1). Firstly, ethyl methanesulfonate (EMS) was used as a mutagenic agent in combination with selection in butanol (EMS + butanol mutants), secondly, EMS was used for mutagenesis but selection was carried out in ethidium bromide (EB) (EMS + EB mutants) and, finally, EB was used as a mutagenic agent and strains were directly selected on agar plates containing EB without exposition to EMS (EB mutants).

For the first two approaches, when EMS was used as a mutagenic agent, a spore suspension of *C. beijerinckii* NRRL B-598 was heated to 80°C for 2 min, vortexed and transferred to Erlenmeyer flasks with TYA medium (Vasylkivska et al., 2019) containing 20 g/L of glucose (analytical reagent grade, PENTA, Chrudim, Czechia). The strain was cultivated in a Concept 400 anaerobic chamber (Ruskinn Technology) under a stable N_2_ atmosphere at 37°C (Figure 1, step 1). After 24 h of cultivation, 2 ml × 2 ml of cell suspension were transferred into sterile Eppendorf tubes, centrifuged and 0.5 ml of fresh sterile TYA medium were added to the cell pellet. Eppendorf tubes were vortexed and 20 μl/ml of EMS (pure, Sigma-Aldrich) were added (Figure 1, step 2). The exposition time was 40 min, during which Eppendorf tubes were placed in the anaerobic chamber. The cell suspension with mutagen was further centrifuged and washed twice with sterile physiological solution. The washed cell suspension was transferred to Petri dishes (250 μl of suspension on each plate) containing HPLC-grade butanol (Sigma-Aldrich) (Figure 1, step A3) or EB (for molecular biology, 10 mg/mL in H_2_O, Sigma-Aldrich) (Figure 1, step B3) for selection. Tolerance of WTS to butanol and EB was tested prior to mutagenesis procedure (details are described in section “Tolerance Testing”) and twice as high a concentration of butanol (for EMS + butanol mutants) or twice as high a concentration of EB (for EMS + EB mutants) than WTS was able to tolerate were used for mutants selection on Petri dishes (Figure 1, step 3). Inoculated petri dishes were cultivated for 48 h at 37°C in the anaerobic chamber. Colonies obtained were transferred to test tubes containing 10 ml of TYA medium with 20 g/L of glucose, and cultivated overnight in the anaerobic chamber (Figure 1, step 4). The cell suspension was cryopreserved in 30% (v/v) glycerol (analytical reagent grade, PENTA, Chrudim, Czechia) solution below −80°C.

EB mutant strains, prepared by direct cultivation on agar plates containing EB without exposition to EMS, were obtained as follows: the spore suspension of *C. beijerinckii* NRRL B-598 was heated to 80°C for 2 min (Figure 1, step C1), vortexed and 50 μl of the spore suspension were transferred onto Petri dishes containing either 0.5 mg/L or 2 mg/L of EB (Figure 1, step C2). Agar plates were cultivated in the anaerobic chamber for 24 h, chosen colonies (Figure 1, step C3) were cultivated in TYA medium and the cell culture was cryopreserved (Figure 1, step 4).

Mutant strains obtained by all methods were tested for butanol tolerance (details are described in section “Tolerance Testing) (Figure 1, step 5). Selected mutant strains with improved butanol tolerance compared to the wild-type strain (WTS) were cultivated in TYA medium (EB mutants) or on TYA agar plates (EMS + butanol and EMS + EB mutants) until sporulation was observed (Olympus BX51 microscope); spore preserves were prepared (Figure 1, step 6). Inocula were prepared from the spore preserves and mutant strains were tested one more time for butanol tolerance (Figure 1, step 7). After this step, the selected strains were further used in the experiments described in this article.

#### Tolerance Testing

Inocula of the wild-type strain and of the mutant strains (except for the first butanol tolerance testing shown as step 5 in Figure 1) were prepared from spore suspensions, and inocula of the mutant strains for the first butanol tolerance testing were prepared from the cryopreserves. Prior to inoculation, the spore suspensions of strains were heated to 80°C for 2 min and vortexed (heat-shock); cryopreserves were thawed at room temperature and vortexed. TYA medium containing 20 g/L of glucose was used for the preparation of inocula and inoculated test tubes were transferred to the anaerobic chamber and cultivated at 37°C overnight.

Tolerance to various substances was tested in microtiter plates containing 120 μl of TYA medium and 10 μl of cell culture. Medium for the experiment additionally contained 0.02 g/L of acid base indicator, bromocresol purple (suitable for indicator, dye content 90%, Sigma-Aldrich), and each substance in different concentrations. Eight substances were tested: two metabolites [butanol in the range of 0 to 30 g/L and ethanol (analytical reagent grade, PENTA, Chrudim, Czechia) in the range of 0 to 65 g/L], efflux pump inducer ethidium bromide EB in the range of 0–6 mg/L and five antibiotics [chloramphenicol (≥98% (HPLC), Sigma-Aldrich] in the range of 0 to 150 mg/L, tetracycline [98.0–102.0% (HPLC), Sigma-Aldrich] in the range of 0–30 mg/L, streptomycin in a form of streptomycin sulfate [>95.0% (TN), Tokyo Chemical Industry] in the range of 0 to 35 mg/L, ampicillin in a form of sodium salt (pure Ph. EUr., AppliChem) in the range of 0–100 mg/L and erythromycin [(for microbiological assay, Sigma-Aldrich) in the range of 0–100 mg/L]. Inoculated microtiter plates were cultivated in the anaerobic chamber at 37°C for 24 h. Results of the testing were evaluated visually as a change in color of the medium, from purple to yellow due to acid production and medium pH shift indicating growth of the strain. For each strain and each substrate, butanol tolerance was tested in at least three repetitions and TYA medium without additions was used as a control.

### Cultivation Experiments

#### Cultivation in Erlenmeyer Flasks

For the determination of glucose consumption rate and metabolite production, WTS and mutant strains were cultivated in triplicates in non-shaken Erlenmeyer flasks. TYA medium containing 40 g/L of glucose was used for both inoculum preparation and cultivation experiment itself. The inoculum was prepared from the spore preserves after heat-shock (as described in section “Tolerance Testing”) and cultivated in an anaerobic chamber under a stable N_2_ atmosphere at 37°C overnight. For the cultivation experiment, flasks were inoculated with 10% (v/v) cell culture and cultivated in the anaerobic chamber for 72 h. At the end of cultivation, samples were taken for pH measurement and subsequent HPLC analysis (details of the analysis are described in section “Analytical Methods”).

Cultivation in triplicates in non-shaken Erlenmeyer flasks with pH control was performed the same way as described above, but TYA medium after sterilization prior to inoculation was supplemented with CaCO_3_ so that concentration 10 g/L of CaCO_3_ was achieved.

#### Cultivation in Bioreactors

According to the result of experiment described in Section “Cultivation in Erlenmeyer Flasks,” one selected mutant strain was chosen for batch cultivation alongside with WTS in triplicates in parallel Multiforce 1L bioreactors (Infors HT).

TYA medium for the inoculum was prepared with glucose concentrations of 20 g/L and for cultivation in bioreactors with concentrations of 40 g/L. The inoculum was prepared from the spore preserves as described in Section “Cultivation in Erlenmeyer Flasks.”. Prior to inoculation of bioreactors, oxygen was exchanged with N_2_ and the pH of the medium was adjusted to 6.4. Bioreactors were inoculated with 10% (v/v) cell culture. Cultivation temperature was 37°C and agitation speed was set to 3.3 s^–1^ throughout the cultivation, the pH was not controlled. During the cultivation, samples were taken for OD measurements and subsequent HPLC analysis (details of the analysis are described in section “Analytical Methods”).

### Analytical Methods

Culture growth was measured as the optical density OD of the culture broth at 600 nm (Varian Cary 50 UV-Vis spectrophotometer, Agilent) against TYA medium as a blank (Vasylkivska et al., 2019).

The concentrations of lactic acid (retention time *t*_*R*_ 6.9 min), acetic acid (*t*_*R*_ 8.1 min), ethanol (*t*_*R*_ 11.7 min), acetone (*t*_*R*_ 12.3 min), butyric acid (*t*_*R*_ 13.5 min), butanol (*t*_*R*_ 24.9 min), and glucose (*t*_*R*_ 4.8 min) were measured using HPLC with refractive index detection (Agilent Series 1200 HPLC). A column with stationary phase of Polymer IEX H from 8 μm (Watrex) was used for the separation. Samples of culture broth were centrifuged and the supernatants were microfiltered. The sample injection volume was 20 μl, the column temperature was 60°C, and 5 mM H_2_SO_4_ was used as the mobile phase with a flow-rate of 1 ml/min. The concentration of substances was determined from calibration curves (Sedlar et al., 2018).

### DNA Isolation and Genome Sequencing

DNA was isolated from exponentially growing cultures prepared by inoculation of TYA medium with heat-shocked spores (as described above) using commercially available isolation kit DNeasy UltraClean Microbial Kit (Qiagen, Hilden, Germany), following recommended instructions. Library construction and sequencing of the sample was performed by CEITEC Genomics core facility (Brno, Czechia) on Illumina NextSeq 500, pair-end, 150 bp.

### Bioinformatics Analysis

The quality assessment of particular steps of data processing were done using FastQC in combination with MultiQC to summarize the reports across all samples (Ewels et al., 2016). Adapter and quality trimming and filtering of singletons was performed with Trimmomatic v0.36 (Bolger et al., 2014). Remaining high quality paired reads were mapped to the genome sequence of the wild type strain with BWA-mem v0.7.15 (Li and Durbin, 2009). The latest genome assembly of the *C. beijerinckii* NRRL B-598 CP011966.3 (Sedlar et al., 2019) was used as a reference. Resulting SAM (Sequence Read Alignment/Map) files were indexed and transformed into more compact BAM (Binary Read Alignment/Map) format using SAMtools v1.9 (Li et al., 2009). Sequences in BAM files were cleaned, sorted, deduplicated and files were indexed with Picard Tools v2.21.6. Single nucleotide variants in genome sequences between wild type strain and particular mutant strains were called with GATK v4.1.4.1 (McKenna et al., 2010). Detected variants were further filtered in order to reduce the number of false positives. For this purpose, WTS resequencing data gathered within the same sequencing run, were used. All variants that were called simultaneously in the WTS and mutant were filtered out. Moreover, we used coverage of false detections in the WTS to set a threshold for filtering, and only variants covered by more than 25.9% of an average coverage of a strain were used. This threshold corresponded to the highest coverage of falsely detected mutations in the WTS. Moreover, not all variants were detected in the whole population. We again set a threshold using analysis of the WTS. Only variants that were called in at least 30.8% of the population were counted. Furthermore, we detected structural variations to the genome sequences, including copy number variations (CNV) with Pilon v 1.22 (Walker et al., 2014). All these analyses were performed with R/Bioconductor using functions from the genomeIntervals v1.42 (Gagneur et al., 2020), Biostrings v2.54 (Pagès et al., 2020), vcfR v1.11 (Knaus and Grünwald, 2017), and ggplot2 (Wickham, 2009) packages.

## Results

### Mutant Strains, Tolerance Testing to Butanol, Ethanol and Tolerance to Known Efflux Pump Substrates

As a first step, WTS *C. beijerinckii* NRRL B-598 was tested for its tolerance to butanol, ethanol and EB. Testing revealed that the strain was able to grow in medium containing no more than 11 ± 1 g/L of butanol, 38.5 ± 2.1 g/L of ethanol or 1.75 ± 0.40 mg/L of EB. EMS + butanol and EMS + EB mutants were, therefore, picked (Figure 1, step 3) from agar plates containing approximately twice as high a concentration of butanol or EB, which were 20 g/L and 4 g/L, respectively, prior to both butanol tolerance testing in microtiter plates (Figure 1, steps 5 and 7).

Number of strains being reduced through screening process is shown in Table 1. For mutagenesis using EMS as mutagenic agent, total of 45 colonies were selected during step A3 (Figure 1) and 45 during B3 (Figure 1). The first butanol tolerance testing (Figure 1, step 5) revealed that 32 strains exhibited increased butanol tolerance compared to WTS. To ensure that increased butanol tolerance was a result of mutation and not adaptation, these 32 strains were cultivated on TYA agar plates until sporulation was observed (Figure 1, step 6) and then spores were germinated and the strains were once again tested for butanol tolerance (Figure 1, step 7). After the second round of butanol tolerance testing, six strains exhibiting increased butanol tolerance were selected for further experiments: EMS + butanol mutants B33 and B44 and EMS + EB mutants E15, E28, E32, and E33 (Table 1).

**TABLE 1:** Number of mutant strains being reduced through screening process^1^.

**Mutagenesis method**	Colonies picked after mutagenesis*	Strains exhibiting increased butanol tolerance (after 1^st^ butanol testing)**	Strains exhibiting increased butanol tolerance (after 2^d^ butanol testing)***	**Strains selected for further experiments**
EB	24	3	3	A, B and C
EMS + butanol	45	13	2	B33 and B44
EMS + EB	45	19	4	E15, E28, E32 and E33

^1^*Steps A3, B3, and C3 from Figure 1; **step 5 from Figure 1; ***step 7 from Figure 1. EB – strains obtained by random chemical mutagenesis using EB as a mutagenic agent, when strains were selected directly on agar plates containing EB; EMS + butanol – strains obtained by random chemical mutagenesis using EMS with selection on butanol; EMS + EB – strains obtained by random chemical mutagenesis using EMS with selection on EB.

Using another approach, mutagenesis of WTS was carried out on agar plates containing EB with no exposition to EMS (EB mutants, Figure 1 steps C1–C3). 24 colonies (Table 1) were picked during C3 step (Figure 1). After the subsequent butanol tolerance test in microtiter plates (Figure 1, step 5), three EB mutants strains exhibited an increase in tolerance, strains A, B, and C. These strains were also cultivated until sporulation was observed (Figure 1, step 6) and then the spores were germinated and the strains were again tested for butanol tolerance (Figure 1, step 7). This confirmed that the acquired increase in tolerance was a stable phenotype.

**FIGURE 2:**
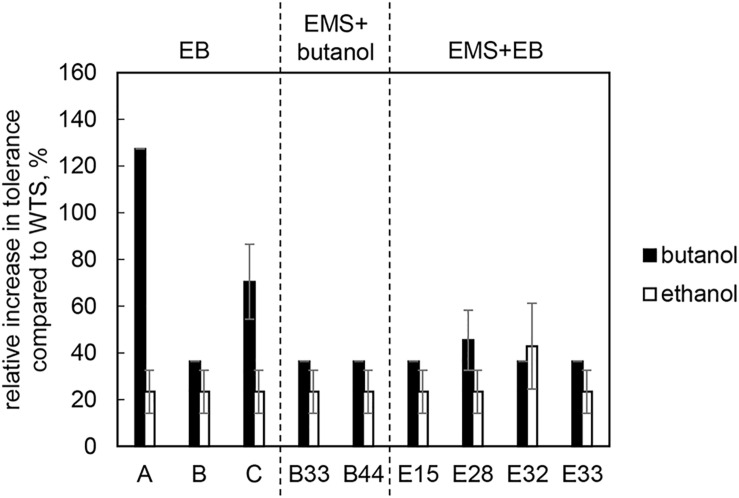
Relative increase in butanol and ethanol tolerance of mutant strains compared to *C. beijerinckii* NRRL B-598 (WTS). Tolerance levels are expressed as the maximum concentration of butanol or ethanol in TYA medium at which growth of the strains was still detectable. The original tolerance of WTS to butanol and ethanol was 11 ± 1 g/L and 38.5 ± 2.1 g/L, respectively. EB – strains obtained by random chemical mutagenesis using EB as a mutagenic agent, when strains were selected directly on the agar plates containing EB; EMS + butanol – strains obtained by random chemical mutagenesis using EMS with selection on butanol; EMS + EB – strains obtained by random chemical mutagenesis using EMS with selection on EB.

All of mutant strains showed a significant increase in butanol and ethanol tolerance compared to WTS (*p* < 0.05, two-sample *t*-test) and were able to grow in a medium containing up to 25.0 g/L of butanol or up to 55.0 g/L of ethanol (Figure 2).

**FIGURE 3:**
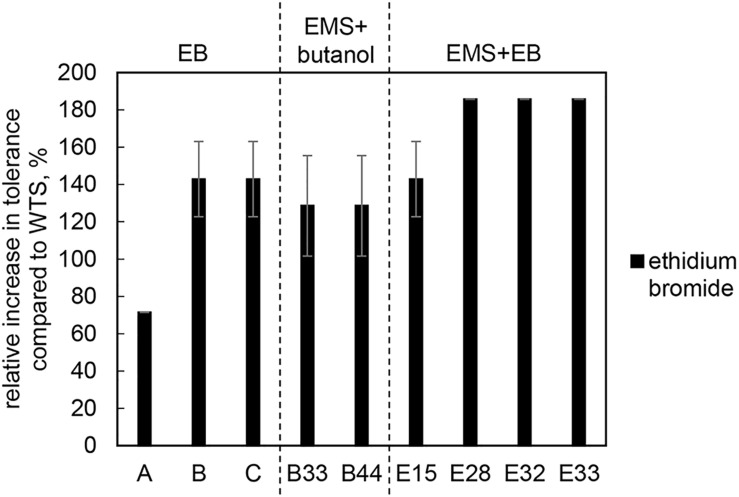
Relative increase in tolerance to EB of mutant strains compared to *C. beijerinckii* NRRL B-598 (WTS). Tolerance levels are expressed as the maximum concentration of substances in TYA medium at which growth of the strains was still detectable. Original tolerance of WTS to EB was 1.75 ± 0.4 mg/L. EB – strains obtained by random chemical mutagenesis using EB as a mutagenic agent, when strains were selected directly on agar plates containing EB; EMS + butanol - strains obtained by random chemical mutagenesis using EMS with selection on butanol; EMS + EB – strains obtained by random chemical mutagenesis using EMS with selection on EB.

As the goal of the study was to increase the efflux capacity of mutant strains, the tolerance of mutant strains and the WTS to different substrates of efflux pumps, i.e., EB and antibiotics, was also tested. Mutants acquired increased EB (up to 5 mg/L) tolerance, see Figure 3.

**FIGURE 4:**
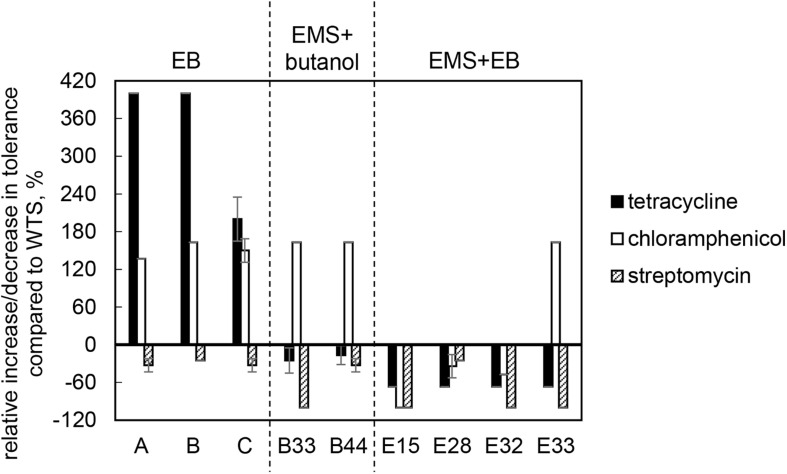
Relative increase/decrease in tolerance to antibiotics tetracycline, chloramphenicol and streptomycin in mutant strains compared to *C. beijerinckii* NRRL B-598 (WTS). Tolerance levels are expressed as the maximum concentration of substances in TYA medium at which growth of the strains was still detectable. Tolerance of WTS was 3.0 ± 0.0, 19.0 ± 1.4, and 20.0 ± 0.0 μg/ml of tetracycline, chloramphenicol and streptomycin, respectively. EB – strains obtained by random chemical mutagenesis using EB as a mutagenic agent, when strains were selected directly on agar plates containing EB; EMS + butanol – strains obtained by random chemical mutagenesis using EMS with selection on butanol; EMS + EB – strains obtained by random chemical mutagenesis using EMS with the selection on EB.

Wild-type strain was tolerant to tetracycline, chloramphenicol and streptomycin at concentrations of 3.0 ± 0.0, 19.0 ± 1.4, and 20.0 ± 0.0 μg/ml, respectively, but was sensitive to ampicillin and erythromycin. The sensitivity toward ampicillin and erythromycin remained unchanged in all mutant strains, but tolerance to tetracycline and chloramphenicol was modified and, in some cases, increased. Surprisingly, tolerance to streptomycin decreased in all cases and strains B33 (EMS + butanol mutant), E15, E32, and E33 (EMS + EB mutants) exhibited complete growth inhibition in the presence of this antibiotic, see Figure 4.

### Metabolites Production by WTS and Mutant Strains

Production of solvents and acids was tested in flasks containing TYA medium. Mutant strains A and C exhibited similar fermentation profiles as WTS and produced butanol in similar concentrations under standard cultivation conditions (Supplementary Table 1). Other mutant strains, B (EB mutant), B33, B44 (EMS + butanol mutants), E15, E28, E32, and E33 (EMS + EB mutants), probably developed the so-called “acid crash” phenotype and their respective fermentation outputs achieved higher concentrations of butyric, acetic or lactic acids compared to WTS.

Inspired by the study of the mutant strain *C. beijerinckii* BA105, which at first was seem to only exhibit the acid crash phenotype but it was later revealed that significantly higher butanol production can be achieved by the strain under pH regulation (Seo et al., 2017), we tested TYA medium with the addition of 10 g/L CaCO_3_ for partial pH control (Table 2). WTS and mutant strains A and C (EB mutants) produced 11–16% higher concentrations of butanol and consumed the total amount of glucose present in the medium. Furthermore, mutant strain B (EB mutant) was able to produce butanol at a concentration of 4.7 ± 0.5 g/L, which is significantly (*p* < 0.05, two-sample *t*-test), almost 12 times, higher than that achieved in the medium without supplementation. However, EMS + butanol and EMS + EB stayed in the acidogenic phase of fermentation and the only difference compared to previous experiments was a significantly (*p* < 0.05, two-sample *t*-test) higher concentration of butyric acid at the end of fermentation.

**TABLE 2:** Concentrations of glucose, acids and solvents and pH reached by *C. beijerinckii* NRRL B-598 (WTS) and its mutant strains in TYA medium containing 10 g/L of CaCO_3_ after 72 h cultivation^1^.

**Mutagenesis method**	**Strain**	**Consumed glucose, g/L**	**Lactic acid, g/L**	**Acetic acid, g/L**	**Ethanol, g/L**	**Acetone, g/L**	**Butyric acid, g/L**	**Butanol, g/L**	**Final pH**
–	WTS	38.4 ± 0.0	0.3 ± 0.0	3.0 ± 0.2	0.4 ± 0.0	1.3 ± 0.1	1.8 ± 0.1	**8.1 ± 0.2**	6.2 ± 0.0
EB	A	38.4 ± 0.0	0.2 ± 0.0	2.5 ± 0.1	0.3 ± 0.0	1.6 ± 0.1	1.7 ± 0.1	**8.1 ± 0.2**	6.4 ± 0.1
EB	B	34.4 ± 1.6	0.2 ± 0.0	3.3 ± 0.5	0.3 ± 0.1	0.1 ± 0.1	9.3 ± 0.7	**4.7 ± 0.5**	5.7 ± 0.0
EB	C	38.4 ± 0.0	0.2 ± 0.0	2.6 ± 0.1	0.4 ± 0.1	1.6 ± 0.1	1.7 ± 0.2	**8.1 ± 0.1**	6.4 ± 0.0
EMS + butanol	B33	26.1 ± 2.0	0.1 ± 0.1	1.5 ± 0.3	0.0 ± 0.0	0.0 ± 0.0	14.2 ± 0.8	**0.0 ± 0.0**	5.6 ± 0.0
EMS + butanol	B44	22.9 ± 0.5	0.1 ± 0.1	1.2 ± 0.0	0.0 ± 0.0	0.0 ± 0.0	12.7 ± 0.3	**0.0 ± 0.1**	5.7 ± 0.0
EMS + EB	E15	26.5 ± 1.1	0.2 ± 0.0	1.0 ± 0.2	0.0 ± 0.0	0.0 ± 0.0	15.2 ± 0.6	**0.0 ± 0.0**	5.7 ± 0.0
EMS + EB	E28	22.4 ± 0.6	0.1 ± 0.1	1.3 ± 0.1	0.0 ± 0.0	0.0 ± 0.0	12.8 ± 0.2	**0.1 ± 0.0**	5.7 ± 0.0
EMS + EB	E32	24.5 ± 1.0	0.2 ± 0.1	1.9 ± 0.2	0.0 ± 0.0	0.0 ± 0.0	13.4 ± 0.4	**0.0 ± 0.0**	5.7 ± 0.0
EMS + EB	E33	26.7 ± 0.8	0.2 ± 0.0	1.7 ± 0.1	0.0 ± 0.0	0.0 ± 0.0	15.0 ± 0.4	**0.0 ± 0.0**	5.7 ± 0.0

**^1^EB, strains obtained by random chemical mutagenesis using EB as a mutagenic agent, when strains were selected directly on agar plates containing EB; EMS + butanol, strains obtained by random chemical mutagenesis using EMS with selection on butanol; EMS + EB, strains obtained by random chemical mutagenesis using EMS with selection on EB*. Bold values are the most important outcomes of the experiment.*

The behavior of the mutant strain with the overall highest butanol production, EB mutant C, was compared with WTS in parallel batch bioreactor fermentation (Supplementary Figure 1). At first sight, the fermentation profiles, growth curves and glucose consumption curves looked very similar, however, a thorough analysis of fermentation data revealed differences in the fermentation dynamics. The pH and growth curves show that the mutant strain C switched to solventogenesis earlier than the WTS and grew faster during the exponential phase. This was also confirmed by calculation of the specific growth rate (μ) for the exponential phase of growth (first 5 h of cultivation as no lag phase was observed), which was 0.32 ± 0.03 h^–1^ for the WTS and 0.48 ± 0.01 h^–1^ for the mutant strain C. Comparison of fermentation parameters for the first 24 h of cultivation and total fermentation time (48 h) is given in Supplementary Table 2. Although the strains reached the same butanol yield (Supplementary Table 2), productivity for mutant C was somewhat higher when calculated for the first 24 h of fermentation, which is in accordance with its higher glucose consumption rate during this period (Supplementary Table 2).

### Genomic Analysis of Mutant Strains

To identify the causes of phenotypic changes in mutant strains, their genomes were sequenced and compared with the WTS genome. After quality trimming and deduplication, 2.4–4.5 million high quality (average Phred score *Q* ≈ 35) paired sequences mapped to the reference genome suggesting coverage from 115 × to 221 × per mutant strain (see Supplementary Table 3). Sequencing of mutant strains revealed that most of the mutations were single-nucleotide polymorphisms (SNP). In unfiltered data, we detected from 21 to 51 SNPs or short indels per mutant strain and 18 false positive detections in the WTS (see Supplementary Files 4). Nevertheless, after filtering (see section “Materials and Methods”), the number of SNPs was reduced to the range from one to 17 per mutant strain (see Table 3). In total, 21 non-synonymous mutations were captured, including a mutation disrupting an open reading frame. SNPs in six genes were observed in at least two mutant strains (Figure 4). SNPs in X276_13415 encoding a putative S-layer family protein was detected in five mutant strains, SNPs in X276_14460 encoding cytochrome b5 was observed in four strains. EB mutants strain A, B and C included SNPs in the same genes, X276_22865 and X276_03000 encoding a carbohydrate ABC transporter permease and an AAA family ATPase, respectively (Figure 4 and Table 3), and no mutations were observed in these gene when EMS was used as the mutagen.

**TABLE 3:** List of single-nucleotide polymorphisms that occurred in mutant strains of *C. beijerinckii* NRRL B-598 exhibiting high butanol tolerance^1^.

	**Position**	**Ref**	**Alt**	**Locus**	**Product**	**Start**	**End**	**Strand**	**Feature**	**Aa_ref**	**Aa_alt**
Mutant strain A*											
1	863781	T	G	X276_22865	Carbohydrate ABC transporter permease	863698	864525	+		I	M
2	1661343	C	T	X276_19395	Peptidase S8 and S53 subtilisin kexin sedolisin	1661151	1662989	+		L	F
3	2775912	T	C	X276_14460	Cytochrome b5	2775169	2775996	+		T	T
4	3007463	C	A	X276_13415	S-layer family protein	3005247	3009269	+		V	V
5	3008654	T	G	X276_13415	S-layer family protein	3005247	3009269	+		T	T
6	5443138	C	T	X276_03000	AAA family ATPase	5441786	5444533	−		E	K
Mutant strain B*											
1	863781	T	G	X276_22865	Carbohydrate ABC transporter permease	863698	864525	+		I	M
2	1190011	G	T						Non-coding		
3	2052101	G	T	X276_17580	Peptidase S8	2051180	2052916	+		G	C
4	3394324	C	T	X276_11885	16S ribosomal RNA	3393103	3394616	−		T	T
5	4087693	C	A						Non-coding		
6	5173544	A	AT	X276_04045	Chemotaxis protein CheC	5173215	5173817	−			
7	5443138	C	T	X276_03000	AAA family ATPase	5441786	5444533	−		E	K
Mutant strain C*											
1	863781	T	G	X276_22865	Carbohydrate ABC transporter permease	863698	864525	+		I	M
2	968295	C	T	X276_22430	Peptidase S8	967488	969206	+		H	Y
3	2775238	G	T	X276_14460	Cytochrome b5	2775169	2775996	+		E	*
4	2919727	T	A	X276_13825	Hypothetical protein	2919158	2919820	+		S	S
5	3004110	C	T	X276_13420	Collagen-like protein	3003304	3004570	+	Pseudogene	G	G
6	3025567	C	A	X276_13335	Collagen-like protein	3025298	3027013	+		G	G
7	4087693	C	A						Non-coding		
8	5443138	C	T	X276_03000	AAA family ATPase	5441786	5444533	−		E	K
9	5649791	C	A						Non-coding		
Mutant strain B33**											
1	81253	G	A	X276_26470	Prolipoprotein diacylglyceryl transferase	81069	81839	+		G	E
2	1115679	G	C						Non-coding		
3	1457462	G	A						Non-coding		
4	1913236	G	A	X276_18195	ABC transporter substrate-binding protein	1912234	1913397	+		D	N
5	3567763	C	T	X276_11100	Methyl-accepting chemotaxis protein	3566625	3568346	−		S	N
6	4087693	C	A						Non-coding		
7	4257246	C	T	X276_07910	DNA polymerase III subunit epsilon	4256489	4257421	−		S	N
8	4560040	C	T	X276_06635	Pyruvate, phosphate dikinase	4560023	4562566	−		V	I
9	5150062	T	A	X276_27350	Hypothetical protein	5146861	5150820	−		T	T
10	5150524	A	T	X276_27350	Hypothetical protein	5146861	5150820	−		S	S
11	5150526	A	T	X276_27350	Hypothetical protein	5146861	5150820	−		S	T
12	5150530	C	T	X276_27350	Hypothetical protein	5146861	5150820	−		V	V
13	5150532	C	T	X276_27350	Hypothetical protein	5146861	5150820	−		V	M
14	5150533	A	T	X276_27350	Hypothetical protein	5146861	5150820	−		I	I
15	5150538	C	T	X276_27350	Hypothetical protein	5146861	5150820	−		V	I
16	5150545	T	C	X276_27350	Hypothetical protein	5146861	5150820	−		G	G
17	5150568	T	C	X276_27350	Hypothetical protein	5146861	5150820	−		I	V
Mutant strain B44**											
1	3006134	C	A	X276_13415	S-layer family protein	3005247	3009269	+		T	T
2	3007658	C	A	X276_13415	S-layer family protein	3005247	3009269	+		G	G
3	4087693	C	A						Non-coding		
Mutant strain E15***											
1	654069	C	T	X276_23670	Nitrogenase iron protein	653549	654403	+		A	V
2	685966	C	T	X276_23545	tRNA 2-thiocytidine biosynthesis protein TtcA	685570	686436	+		P	S
3	724396	GT	G						Non-coding		
4	1809500	CA	C	X276_18715	YggS family pyridoxal phosphate-dependent enzyme	1809001	1809681	+			
5	2376845	G	A	X276_16220	MFS transporter	2376293	2377702	−		Y	Y
6	2453563	G	A	X276_15915	Sigma-54-dependent Fis family transcriptional regulator	2451932	2453896	+		E	E
7	2468045	G	A	X276_15855	HlyC/CorC family transporter	2467109	2468398	+		A	T
8	2482557	C	T	X276_15775	Bifunctional 4-hydroxy-2-oxoglutarate aldolase/2-dehydro-3-deoxy-phosphogluconate aldolase	2482286	2482915	+		A	V
9	3008051	C	A	X276_13415	S-layer family protein	3005247	3009269	+		T	T
10	3141441	C	CA	X276_12855	DUF4179 domain-containing protein	3141433	3142683	+			
11	4087693	C	A						Non-coding		
12	4243433	C	CT	X276_07980	MarR family transcriptional regulator	4243239	4243676	−			
Mutant strain E28***											
1	491837	G	A	X276_24400	MerR family transcriptional regulator	491399	492223	+		E	K
2	851273	T	G	X276_22910	16S ribosomal RNA	851024	852535	+		*	G
3	851321	T	G	X276_22910	16S ribosomal RNA	851024	852535	+		*	G
4	2775807	C	A	X276_14460	Cytochrome b5	2775169	2775996	+		G	G
5	2775912	T	C	X276_14460	Cytochrome b5	2775169	2775996	+		T	T
6	3004250	C	A	X276_13420	Collagen-like protein	3003304	3004570	+	Pseudogene	P	Q
7	3007799	C	A	X276_13415	S-layer family protein	3005247	3009269	+		T	T
8	3008654	T	G	X276_13415	S-layer family protein	3005247	3009269	+		T	T
Mutant strain E32***											
1	2207690	C	CT	X276_16910	IS110 family transposase	2206548	2207848	−	Pseudogene		
2	2775912	T	C	X276_14460	Cytochrome b5	2775169	2775996	+		T	T
3	3007397	C	A	X276_13415	S-layer family protein	3005247	3009269	+		G	G
4	3876973	C	A	X276_09805	Glycoside hydrolase	3876787	3877821	−		M	I
5	4087693	C	A						Non-coding		
6	4243379	C	CAT	X276_07980	MarR family transcriptional regulator	4243239	4243676	−			
7	4960470	G	A	X276_04930	Chemotaxis protein	4958594	4963015	−		T	I
8	4982628	C	T	X276_04840	Response regulator	4982393	4982745	−	Pseudogene	I	I
9	5011570	C	T	X276_04700	Non-ribosomal peptide synthase	5010682	5018304	−		W	*
10	5053669	C	T	X276_04620	Hypothetical protein	5053325	5055736	−		D	N
11	5083112	C	T	X276_04450	YigZ family protein	5082972	5083619	−		V	I
12	5084395	C	T	X276_04445	PLP-dependent aminotransferase family protein	5083996	5085438	−		M	I
13	5275399	G	A	X276_03630	Carboxynorspermidine decarboxylase	5274563	5275702	−		H	Y
Mutant strain E33***											
1	2663675	G	A	X276_14895	PFL family protein	2663567	2664922	+		G	R

^1^Ref, DNA base in the wild-type strain; Alt, DNA base in mutant strain; Start, position of the gene in the genome, start; End, position of the gene in the genome, end; Aa_ref, encoded amino acid in the wild-type strain; Aa_alt, encoded amino acid in mutant strain. *Strains obtained by random chemical mutagenesis using EB as a mutagenic agent, when strains were selected directly on agar plates containing EB; **strains obtained by random chemical mutagenesis using EMS with selection on butanol; ***strains obtained by random chemical mutagenesis using EMS with selection on EB.

In addition to SNPs, we detected several longer genome changes, including copy number variations (CNVs) (Tables 4, 5). No copy number variations were detected for mutant strain E32. CNVs were detected for similar positions in strains A, B. C, B44, E28, and E33 (Table 5).

**TABLE 4:** List of mutations that caused longer changes in the genomes of mutant strains of *C. beijerinckii* NRRL B-598 exhibiting high butanol tolerance^1^.

**Strain**	**Mutation position**	**Size (bp)**	**Note**
B33*	5311613-5311767	155	Intergenic
E15**	2982416-2982635	220	Part of the gene X276_13505 encoding DNA mismatch repair protein MutS
	5311613-5311767	155	Intergenic
E33**	4243635-4244393	759	Part of the gene X276_07980 encoding MarR family transcriptional regulator

^1^*Strains obtained by random chemical mutagenesis using EMS with the selection on butanol; **strains obtained by the random chemical mutagenesis using EMS with selection on ethidium bromide.

**TABLE 5:** List of copy number variations in the genomes of mutant strains of *C. beijerinckii* NRRL B-598 exhibiting high butanol tolerance^1^.

**Strain**	**Position**	**Size**	**Locus**	**Putative product**
A*	341278–349235	7.96 kb	X276_25195	DNA-3-methyladenine glycosylase 2 family protein
			X276_25190	Site-specific integrase
			X276_25185	XRE family transcriptional regulator
			X276_25180	Hypothetical protein
			X276_25175	Transcription factor
			X276_25170	Hypothetical protein
			X276_25165	Replication protein
			X276_25160	Hypothetical protein
			X276_25155	Hypothetical protein
			X276_25150	Hypothetical protein
			X276_25145	Hypothetical protein
B*	341243–349259	8.02 kb	X276_25195	DNA-3-methyladenine glycosylase 2 family protein
			X276_25190	Site-specific integrase
			X276_25185	XRE family transcriptional regulator
			X276_25180	Hypothetical protein
			X276_25175	Transcription factor
			X276_25170	Hypothetical protein
			X276_25165	Replication protein
			X276_25160	Hypothetical protein
			X276_25155	Hypothetical protein
			X276_25150	Hypothetical protein
			X276_25145	Hypothetical protein
C*	341297–349217	7.92 kb	X276_25195	DNA-3-methyladenine glycosylase 2 family protein
			X276_25190	Site-specific integrase
			X276_25185	XRE family transcriptional regulator
			X276_25180	Hypothetical protein
			X276_25175	Transcription factor
			X276_25170	Hypothetical protein
			X276_25165	Replication protein
			X276_25160	Hypothetical protein
			X276_25155	Hypothetical protein
			X276_25150	Hypothetical protein
			X276_25145	Hypothetical protein
B33**	167230–167409	180 bp	X276_26025	PIN/TRAM domain-containing protein
	499439–499573	135 bp	Only intergenic	–
	575045–575230	186 bp	Only intergenic	–
	728816–728957	142 bp	X276_23400	Hypothetical protein
	6042705–6042895	191 bp	X276_00695	Methyl-accepting chemotaxis protein
	6137871–6138337	467 bp	X276_00305	23S rRNA [pseudouridine(1915)-N(3)]-methyltransferase RlmH
B44**	341237–349270	8.03 kb	X276_25195	DNA-3-methyladenine glycosylase 2 family protein
			X276_25190	Site-specific integrase
			X276_25185	XRE family transcriptional regulator
			X276_25180	Hypothetical protein
			X276_25175	Transcription factor
			X276_25170	Hypothetical protein
			X276_25165	Replication protein
			X276_25160	Hypothetical protein
			X276_25155	Hypothetical protein
			X276_25150	Hypothetical protein
			X276_25145	Hypothetical protein
E15***	4901–5166	266 bp	X276_26795	DNA topoisomerase (ATP-hydrolyzing) subunit B
E28***	341278–349256	7.98 kb	X276_25195	DNA-3-methyladenine glycosylase 2 family protein
			X276_25190	Site-specific integrase
			X276_25185	XRE family transcriptional regulator
			X276_25180	Hypothetical protein
			X276_25175	Transcription factor
			X276_25170	Hypothetical protein
			X276_25165	Replication protein
			X276_25160	Hypothetical protein
			X276_25155	Hypothetical protein
			X276_25150	Hypothetical protein
			X276_25145	Hypothetical protein
E33***	341393–349134	7.74 kb	X276_25190	Site-specific integrase
			X276_25185	XRE family transcriptional regulator
			X276_25180	Hypothetical protein
			X276_25175	Transcription factor
			X276_25170	Hypothetical protein
			X276_25165	Replication protein
			X276_25160	Hypothetical protein
			X276_25155	Hypothetical protein
			X276_25150	Hypothetical protein
			X276_25145	Hypothetical protein

^1^*Strains obtained by random chemical mutagenesis using EB as a mutagenic agent, when strains were selected directly on agar plates containing EB; **strains obtained by random chemical mutagenesis using EMS with selection on butanol; ***strains obtained by random chemical mutagenesis using EMS with selection on EB.

## Discussion

Using three different approaches of random chemical mutagenesis, we were able to obtain nine mutant strains of *C. beijerinckii* NRRL B-598 exhibiting from 36 to 127% increases in butanol tolerance (Figure 2). Similar increases in tolerance have been described for other solventogenic clostridia; in fact, the most well-studied and best-performing mutant strain, *C. beijerinckii* BA101, was likewise obtained by random chemical mutagenesis (Annous and Blaschek, 1991). *C. beijerinckii* BA101, an offspring of *C. beijerinckii* NCIMB 8052, exhibited around a 110% increase in butanol tolerance (Qureshi and Blaschek, 2001). Similarly, the asporogenic mutant *C. beijerinckii* ATCC 55025, an offspring of *C. acetobutylicum* ATCC 4259, was able to tolerate about 11.4 g/L of butanol while its WTS only 5 g/L (Jain et al., 1994). Interestingly, two of our nine mutant strains of *C. beijerinckii* NRRL B-598 with the highest increase in butanol tolerance were obtained by mutagenesis on agar plates containing EB (EB mutants) (Figure 2), a method that has not previously been used for this purpose in solventogenic *Clostridium*.

Despite increased butanol tolerance, no mutant strain exhibited an increase in butanol production compared with WTS (Supplementary Table 1 and Table 2), which is a divergence from well-known mutant strains obtained in a similar way. For example, *C. beijerinckii* BA101 was able to produce around 20 g/L of butanol during batch cultivation (an increase of over 100%) (Annous and Blaschek, 1991; Chen and Blaschek, 1999) and *C. beijerinckii* ATCC 55025 displayed an increase in butanol concentration between 22 and 38% (Jain et al., 1994). However, it has previously been shown in multiple studies, both for random (Baer et al., 1987; Gallardo et al., 2017; Máté de Gérando et al., 2018) and targeted (Zhao et al., 2003; Alsaker et al., 2004; Mann et al., 2012; Jones et al., 2016) mutagenesis, that increased butanol tolerance does not always result in improved butanol production.

EMS + butanol and EMS + EB mutant strains exhibited the acid crash phenotype under standard cultivation conditions and under pH regulation via CaCO_3_ supplementation (Supplementary Table 1 and Table 2), producing high concentrations of butyric acid. It was shown for the WTS that it is possible to cultivate the strain at larger scale under constant pH regulation to produce butyric acid as the main fermentation product (Drahokoupil and Patáková, 2020). Therefore, these mutant strains can be further studied as alternative butyric acid producers.

Along with higher butanol tolerance, mutant strains of *C. beijerinckii* NRRL B-598 exhibited improved tolerance to ethanol and EB (Figures 2, 3). Tolerance testing revealed that mutant strains obtained by different approaches behaved differently. While tolerance to ethanol increased similarly in all mutant strains (Figure 2), EMS + EB mutants exhibited higher tolerance to EB than other strains (Figure 3). In the case of antibiotics, EB mutants generally exhibited higher tolerance (Figure 4). On the other hand, antibiotic tolerance of EMS + EB strains was lower than the WTS, except for strain E33 when tested for chloramphenicol (Figure 4). These differences can be probably explained by different mutations that occurred in the strains, so to reveal the differences at the gene level, genomes of all mutant strains were sequenced and variant callings were analyzed.

Variant calling in bacteria is a neglected topic and mainly, attention is paid to eukaryotes. Nevertheless, one of the first benchmarking studies dealing with bacterial variant calling by Bush et al. (2020) showed that a combination of BWA for mapping and GATK HaplotypeCaller brought the best results for closely related strains. As this was the case in our study, we used these tools to capture SNPs (Supplementary Files 4). Moreover, we took advantage of our data containing resequencing of the WTS that was recently used to update its genome assembly (CP011966.3) (Sedlar et al., 2019). We used false positive detections of SNPs in the WTS sequencing data to infer our own filtering rules (see section “Materials and Methods”) (Table 3). While this approach is good for SNP detection, it is unable to call structural variants. Thus, we utilized Pilon to detect longer changes in genomic sequences (Tables 4, 5). The combination of various approaches for detection of short and longer changes is quite common for eukaryotes (Long et al., 2013). Utilization of Pilon is advantageous in our case as the reference sequence was constructed using this approach, and thus, we were again able to remove false positives by filtering variants that were falsely called in the WTS data. SNPs in individual genes of the mutants are discussed further in the text, however, it is difficult to discuss CNVs because the topic has been scarcely studied in bacteria. CNVs are usually studied in comparative analyses (Větrovský and Baldrian, 2013; Greenblum et al., 2015) and the approach cannot be applied in our case.

**FIGURE 5:**
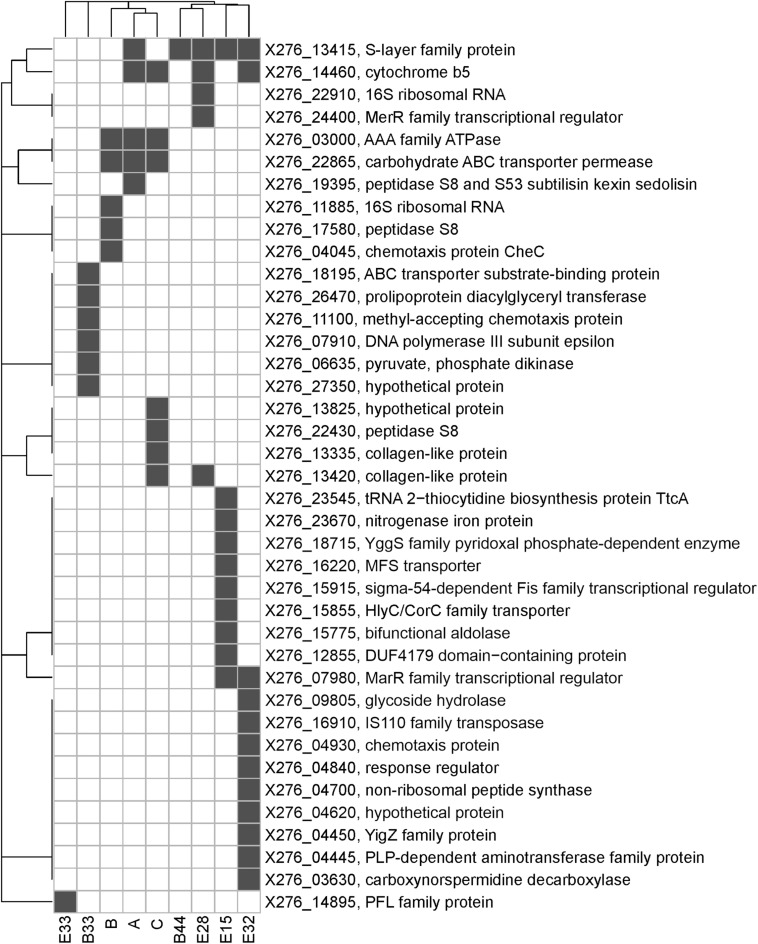
A bi-clustered binary heatmap showing the distribution of genes with single-nucleotide polymorphisms among mutant strains of *C. beijerinckii* NRRL B-598 with improved butanol tolerance. Complete linkage clustering is based on binary distance between genes and mutants, respectively. Strains A, B, and C were obtained by random chemical mutagenesis using EB as a mutagenic agent, when strains were selected directly on agar plates containing EB; Strains B33 and B44 were obtained by random chemical mutagenesis using EMS with selection on butanol; strains E15, E28, E32, and E33 were obtained by random chemical mutagenesis using EMS with selection on EB.

Only few genome sequences of butanol-tolerant mutant strains obtained by random mutagenesis are currently available [*C. beijerinckii* SA-1 (Sandoval-Espinola et al., 2013), *C. pasteurianum* M150B (Sandoval et al., 2015), *C. acetobutylicum* ATCC 55025 and *C. acetobutylicum* JB200 (Xu et al., 2017)]. Genomic sequences of *C. acetobutylicum* ATCC 55025 and *C. acetobutylicum* JB200, as well as *C. pasteurianum* M150B, included multiple mutations, for example, 143 SNPs and 67s SNP for *C. acetobutylicum* ATCC 55025 and *C. pasteurianum* M150B, respectively. On the other hand, similar to the case of *C. beijerinckii* SA-1, for which 10 genetic polymorphisms were confirmed, including eight SNPs, genomes of our mutant strains of *C. beijerinckii* NRRL B-598 included one to 17 SNPs (21–51 prior to filtering), one longer change in strains B33, E15, and E33 and one or more copy number variations in all of the strains, except E32 (Tables 3–5). Some of the mutations revealed in *C. beijerinckii* NRRL B-598 mutant strains with increased butanol tolerance can be connected to tolerance mechanisms, while others may play roles in metabolic enhancement rather than tolerance improvement. Many of the mutations were membrane-related, which correlates with the fact that normal cellular responses of the strains to the solvent was mainly at the membrane level (Patakova et al., 2018). A similar result was observed in *E. coli* when a genomic library enrichment strategy was used under butanol challenge (Reyes et al., 2011). Examples of membrane-related mutations in *C. beijerinckii* NRRL B-598 mutant strains are mutations in genes encoding peptidases, transporters or cytochrome b5 (Table 3). Interestingly, mutations in genes encoding peptidase S8 were observed not only in our EB mutants A (X276_19395), B (X276_17580), and C (X276_22430) (Table 3), but also in a mutant strain of *C. beijerinckii* SA-1, the offspring of *C. beijerinckii* NCIMB 8052 (Sandoval-Espinola et al., 2013). Additionally, it seems that EB mutants A, B and C not only exhibited different phenotypes from other mutants for which EMS was used as a mutagenic agent, but were also different in terms of mutations and formed a distinguishable cluster, as shown in Figure 5.

We analyzed changes in the genomes of mutant strains to determine whether some of the mutations occurring in EMS + butanol and EMS + EB strains could lead to the acid-crash phenotype. In the case of strain E15, the mutation occurred in gene X276_15915 encoding the sigma-54-dependent Fis family transcriptional regulator (Table 3). This may be causing the acid crash phenotype. Sigma-54 regulates sugar consumption and carbon metabolism in *C. beijerinckii* (Hocq et al., 2019). Thus, a higher glucose uptake rate and acid production rate, the reasons for the acid crash phenotype (Maddox et al., 2000), may occur due to mutations in regulatory genes such as sigma-54. A similar hypothesis has already been proposed for the degenerate strain *C. beijerinckii* DG-8052 (Lv et al., 2016). Unfiltered data contained a mutation that might be responsible for the phenotype; it is situated in gene X276_15350 encoding NADP-dependent glyceraldehyde-3-phosphate dehydrogenase in mutant strain E28 (Supplementary Files 4). The gene product plays a role in the formation of NADPH, which is necessary for the biosynthetic processes and modulation of redox potential (Liu et al., 2015). The importance of the gene for metabolite production was shown when expression of NADP-dependent glyceraldehyde-3-phosphate dehydrogenase of *C. acetobutylicum* in *E. coli* resulted in improved productivity of lycopene and ε-caprolactone (Martínez et al., 2008). As in the case of solventogenic strain *C. saccharoperbutylacetonicum*, where defects in genes encoding enzymes responsible for NADH formation resulted in strain degeneration (Hayashida and Yoshino, 1990), mutations in genes encoding NADPH formation in mutant strain E28 could affect the cells in a similar way. Nevertheless, this mutation (in gene X276_15350) was later filtered (Table 3) and, therefore, acid crash in the E28 strain probably happened due to some other changes. The reason for the acid crash phenotype in other mutant strains was not clear and needs further investigation.

The faster growth and somewhat higher butanol productivity of mutant C, observed during the first 24 h of cultivation (Supplementary Table 2), can probably be attributed to mutations in genes encoding a carbohydrate ABC transporter permease (X276_22865) and an AAA family ATPase (X276_03000), in which mutations were also detected for the other EB mutants, A and B (Figure 5). Use of non-PTS mechanisms of glucose uptake during the solventogenic phase, such as ABC transporters, resulted in more complete glucose utilization and increased butanol production in *C. beijerinckii* BA101 (Lee et al., 2005). A BLASTp (Gish and States, 1993) analysis of AAA family ATPase X276_03000 showed that it contained motifs similar to the PTS operon transcription anti-terminator in *Clostridioides difficile* 630, therefore it probably plays a part in PTS regulation. It was reported that the genome of the butanol-overproducing mutant *C. beijerinckii* SA-1 included mutations in PTS genes (Sandoval-Espinola et al., 2013) and that the mutant strain *C. beijerinckii* BA101 had a partially defective PTS (Lee and Blaschek, 2001; Lee et al., 2005). Therefore, a connection between PTS and regulation of solvent production, contributing to butanol overproduction, was hypothesized (Sandoval-Espinola et al., 2013).

Mutations in genes connected to butanol tolerance can also be identified in mutant strains of *C. beijerinckii* NRRL B-598. For example, in genes encoding chemotaxis proteins in strains B (X276_04045), B33 (X276_11100 and X276_00695), and E32 (X276_04930) (Tables 3, 5). Chemotaxis is one the important mechanisms of adaptation to environmental stress (Zhao et al., 2007), in our case, the presence of produced butanol in the medium. It was shown that butanol acts as a repellent for *C. acetobutylicum*, meaning that it induces negative chemotaxis in the strain (Gutierrez and Maddox, 1987). For strain E32, mutations were observed in the gene X276_09805 encoding glycoside hydrolase, which belongs to family 25 (Table 3). This family includes enzymes with lysozyme activity and ones connected to autolysin production, for example, lyc gene (CA_C0554) of *C. acetobutylicum* ATCC 824. It was reported that enzymes that take part in autolysis and cell wall recycling in solventogenic clostridia are also connected to butanol tolerance (Patakova et al., 2018). In mutant strain B33, mutations were detected in X276_26470 encoding prolipoprotein diacylglyceryl transferase (Table 3), which catalyzes attachments of lipoproteins to cell membrane. Lipoproteins take part in multiple processes in the cell, including membrane transport, modifications of the cell wall, and antibiotic tolerance (Nielsen and Lampen, 1982; Chimalapati et al., 2012); therefore, mutations in prolipoprotein diacylglyceryl transferase can also be connected to butanol tolerance mechanisms.

One of most interesting mutations connected to improved butanol tolerance was revealed in EMS + EB mutants E15, E32, and E33 in the gene X276_07980 encoding the MarR family transcriptional regulator (Tables 3, 4). Similarly to our result, mutations in genes encoding the MarR family transcriptional regulator occurred for the hyper butanol-tolerant and -producing strain, *C. acetobutylicum* JB200 and the authors hypothesized that this mutation contributed to enhanced butanol tolerance (Xu et al., 2017). Interestingly, MarR is a regulator that contributes to antibiotic resistance, as well as resistance to organic solvents and oxidative stress agents, by modulating the efflux pump and porin expression (Sharma et al., 2017). Thus, other regulators may also contribute to enhanced butanol tolerance. For example, mutations in gene X276_24400 encoding the MerR family transcriptional regulator in mutant strain E28 (Table 3). A BLASTp (Gish and States, 1993) analysis showed that the MerR gene shares similarity with the BmrR regulator from *Bacillus subtilis*, which controls the Bmr efflux pump for removal of antibiotics, dyes and disinfectants (Ahmed et al., 1994). Therefore, mutations in efflux pump regulators were found in all EMS + EB mutants.

These results suggest that the increased or decreased tolerance of mutant strains to antibiotics, EB or solvents (butanol and ethanol) (Figures 2–4) may also be due to altered activities of efflux pumps controlled by regulators. Transcriptional analysis of *C. beijerinckii* NRRL B-598 revealed upregulation of the TetR/AcrR family regulators putatively involved in efflux pump gene transcription after butanol addition to the medium (Sedlar et al., 2019), suggesting that efflux pumps might be involved in overcoming butanol stress in the strain. In the *C. beijerinckii* NRRL B-598 genome, 55 genes were identified as putatively encoding efflux pumps (Jureckova et al., 2018). Some of these pumps may be able to remove antibiotics, EB or solvents from a cell. For example, genes encoding Gram-positive efflux pumps capable of transporting tested antibiotics have been described for *Streptococcus pneumoniae*, *B. subtilis*, *Enterococcus faecalis*, *Lactococcus lactis*, and *Corynebacterium glutamicum* (Schindler and Kaatz, 2016). An efflux pump responsible for increased ethanol tolerance was reported for *Saccharomyces cerevisiae* BY4741 (Yang et al., 2013). Furthermore, native efflux pumps able to actively remove butanol were recently discovered in *Pseudomonas putida* and *E. coli* (Basler et al., 2018; Zhang et al., 2018), and heterologous expression of such a butanol efflux pump from *P. putida* in butanol-producing *C. saccharoperbutylacetonicum* led to increased butanol tolerance of the strain (Jiménez-Bonilla et al., 2020).

## Conclusion

Our study shows that random chemical mutagenesis using EB can be successfully used for the generation of butanol-tolerant mutant strains in solventogenic *Clostridium*. While mutagenesis with EMS as a mutagenic agent, which is more commonly used for such a purpose, resulted in mutants exhibiting the acid-crash phenotype, use of EB alone did not disrupt solvent production. Moreover, use of EB for both mutagenesis and selection resulted in increased tolerance to several different substrates of efflux pumps.

We speculate that the acid crash phenotype in mutant strain E15 was acquired due to mutations in the gene encoding the sigma-54-dependent Fis family transcriptional regulator. Further investigations are needed to reveal the reasons for the phenotype in other EMS + butanol and EMS + EB strains.

Higher butanol tolerance of the strains may be connected to mutations in genes connected to the stress response, for example, glycoside hydrolase or prolipoprotein diacylglyceryl transferase. However, the most prominent change in tolerance to substrates of efflux pumps, including butanol, can be explained by mutations in genes encoding efflux pump regulators, which were found in EMS + EB mutants. These regulators can be further studied in research connected to butanol tolerance mechanisms using targeted mutagenesis.

## Data Availability Statement

The genome sequencing data have been deposited in the NCBI Sequence Read Archive (SRA) under the project accession number PRJNA229510 (https://www.ncbi.nlm.nih.gov/sra/?term=PRJNA229510). WTS data are available under accession number SRX6419139 and mutant strains data under accession number within the range from SRX8614691 to SRX8614699.

## Author Contributions

MV, KS, IP, and PP designed the study. MV and BB performed the experiments and analyzed the data. KS and KJ performed the bioinformatics analyses. MV wrote the original draft, KS, BB, and PP contributed to manuscript writing. All authors read and approved the final manuscript.

## Conflict of Interest

The authors declare that the research was conducted in the absence of any commercial or financial relationships that could be construed as a potential conflict of interest.
