# Supplementary material for: Phenotypic and Genomic Analysis of Clostridium beijerinckii NRRL B-598 Mutants With Increased Butanol Tolerance
Source: Front Bioeng Biotechnol. 2020 Nov 5;8:598392. doi: 10.3389/fbioe.2020.598392 (PMC7674653; doi:10.3389/fbioe.2020.598392)
Supplement: Supplementary file 1 [file Data_Sheet_1.DOCX]

Supplementary Material

**Supplementary Table 1.** Concentrations of glucose, acids and solvents, and pH achieved by *C. beijerinckii* NRRL B-598 (WTS) and its mutant strains in TYA medium containing 40 g/L of glucose after 72 h cultivation.^^[[1]](#footnote-1)^^

| **Mutagenesis method** | **Strain** | **Consumed glucose, g/L** | **Lactic acid, g/L** | **Acetic acid, g/L** | **Ethanol, g/L** | **Acetone, g/L** | **Butyric acid, g/L** | **Butanol, g/L** | **Final pH** |
| --- | --- | --- | --- | --- | --- | --- | --- | --- | --- |
| - | WTS | 29.1±1.1 | 0.4±0.1 | 1.2±0.0 | 0.2±0.0 | 1.1±0.0 | 1.6±0.0 | **7.3±0.1** | 5.3±0.1 |
| EB | A | 30.4±3.7 | 0.2±0.1 | 1.1±0.1 | 0.2±0.0 | 1.5±0.2 | 0.8±0.1 | **7.0±0.8** | 5.8±0.1 |
| EB | B | 5.9±1.2 | 0.8±0.0 | 2.0±0.1 | 0.0±0.0 | 0.0±0.0 | 3.6±0.3 | **0.4±0.1** | 4.9±0.0 |
| EB | C | 34.4±0.5 | 0.3±0.0 | 1.1±0.0 | 0.2±0.0 | 1.9±0.1 | 0.9±0.0 | **7.3±0.2** | 6.0±0.1 |
| EMS + butanol | B33 | 10.1±2.1 | 1.0±0.1 | 1.6±0.1 | 0.0±0.0 | 0.0±0.0 | 3.2±0.2 | **0.1±0.0** | 4.9±0.0 |
| EMS + butanol | B44 | 7.6±0.6 | 1.1±0.2 | 1.8±0.1 | 0.0±0.0 | 0.0±0.0 | 3.3±0.3 | **0.1±0.0** | 4.9±0.0 |
| EMS + EB | E15 | 6.9±0.2 | 0.7±0.2 | 2.0±0.4 | 0.0±0.0 | 0.0±0.0 | 3.9±0.0 | **0.1±0.0** | 4.9±0.0 |
| EMS + EB | E28 | 7.3±1.0 | 0.6±0.2 | 1.9±0.1 | 0.0±0.0 | 0.0±0.0 | 4.3±0.6 | **0.1±0.1** | 4.9±0.0 |
| EMS + EB | E32 | 7.0±0.2 | 0.8±0.1 | 2.9±0.2 | 0.0±0.0 | 0.0±0.0 | 3.3±0.1 | **0.1±0.0** | 4.8±0.0 |
| EMS + EB | E33 | 7.4±0.4 | 0.5±0.0 | 1.7±0.0 | 0.0±0.0 | 0.0±0.0 | 4.2±0.1 | **0.1±0.0** | 5.0±0.0 |


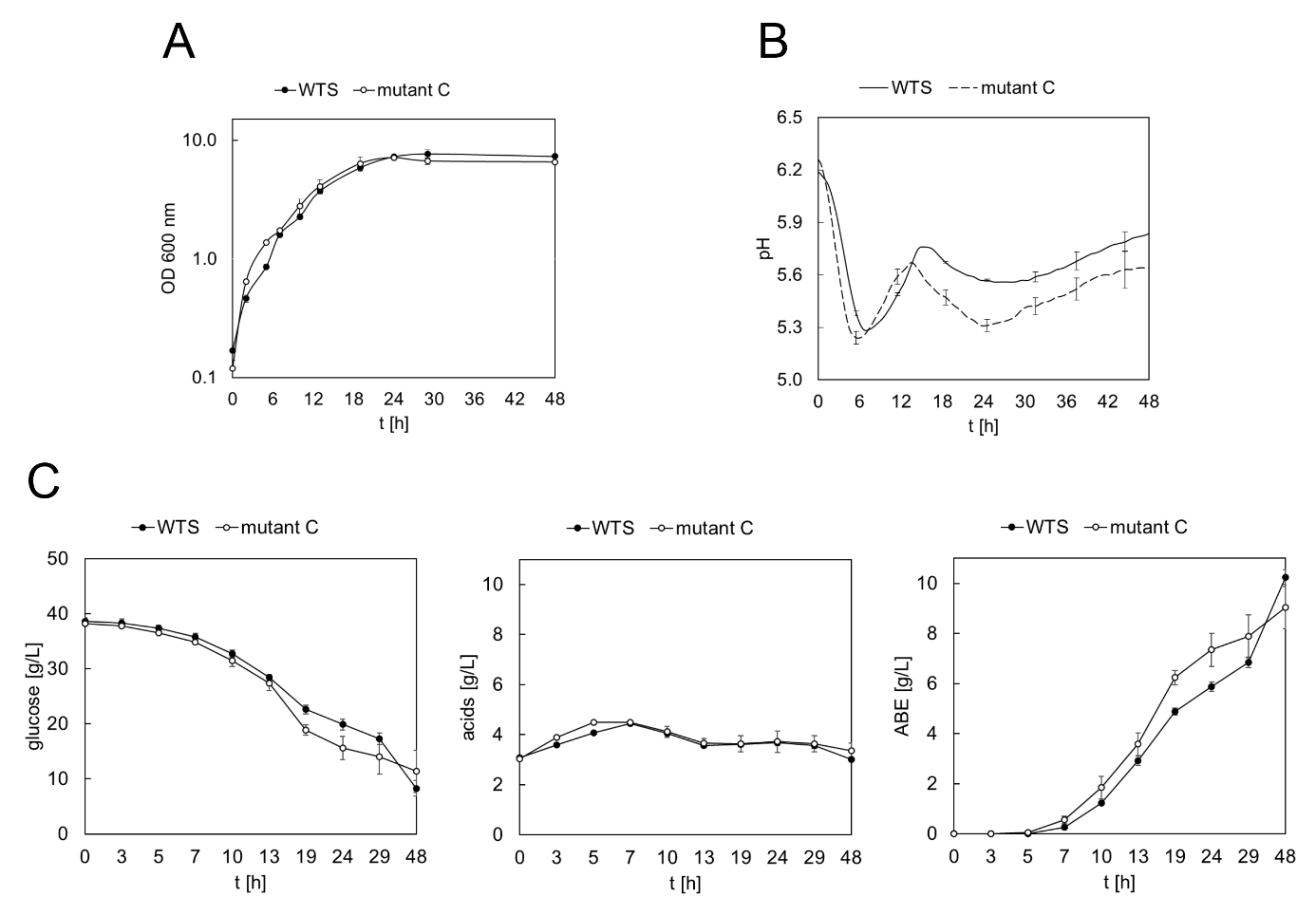


**Supplementary Figure 1.** Batch cultivation characteristics of *C. beijerinckii* NRRL B-598 (WTS) and its mutant strain C with increased butanol tolerance in TYA medium. **(A)** Growth curve. **(B)** Time course of pH change. **(C)** Concentrations of glucose, acids and the total concentration of acetone, butanol and ethanol (ABE) over the time course of cultivation. Error bars represent standard deviations.

**Supplementary Table 2.** Yield, butanol productivity and glucose consumption during batch bioreactor cultivation of *C. beijerinckii* NRRL B-598 (WTS) and its mutant strain C with higher butanol tolerance, calculated for the first 24 h of cultivation and at the end of cultivation (48 h). ^^[[2]](#footnote-2)^^

| Parameter | Strain | 24 h | 48 h |
| --- | --- | --- | --- |
| Yield  (g butanol/g glucose) | WTS | 0.19±0.00 | 0.20±0.00 |
|  | Mutant strain C | 0.19±0.01 | 0.20±0.00 |
| Butanol production rate (g/L/h): | WTS | 0.15±0.01 | 0.13±0.01 |
|  | Mutant strain C | 0.18±0.02 | 0.11±0.01 |
| Glucose consumption rate (g/L/h): | WTS | 0.78±0.02 | 0.63±0.02 |
|  | Mutant strain C | 0.94±0.07 | 0.56±0.07 |

**Supplementary Table 3.** Sequencing coverage of *C. beijerinckii* NRRL B-598 (WTS) and its mutant strains’ genomes

| **Strain** | **No. of reads**^^[[3]](#footnote-3)^^ | **Coverage** |
| --- | --- | --- |
| WTS | 7980255 | 193× |
| A | 6563393 | 159× |
| B | 7596983 | 184× |
| C | 7619524 | 184× |
| B33 | 4751416 | 115× |
| B44 | 6730003 | 163× |
| E15 | 8952110 | 217× |
| E28 | 8612344 | 209× |
| E32 | 9104809 | 221× |
| E33 | 8111651 | 197× |

1. EB – strains obtained by random chemical mutagenesis using ethidium bromide (EB) as a mutagenic agent, when strains were selected directly on agar plates containing EB; EMS+butanol - strains obtained by random chemical mutagenesis using ethyl methanesulfonate (EMS) with selection on butanol; EMS+EB - strains obtained by random chemical mutagenesis using EMS with selection on EB. [↑](#footnote-ref-1)
2. Specific growth rate was calculated using the following equation:

$$\mu=\text{ }\frac{\ln X_{2}-lnX_{1}}{t_{2}-t_{1}} \left( h^{-1} \right),$$

   where X_1_ represents biomass concentration (expressed as OD_600_) at the beginning of the exponential phase, X_2,_ the biomass concentration (expressed as OD_600_) at the end of the exponential phase, and t_1_ and t_2_ are cultivation times at the beginning and the end of the exponential phase respectively.

   Butanol yield on glucose was calculated using the following equation:

$$Y_{P/S}=\frac{P_{\mathrm{final}}-P_{0}}{S_{0}-S_{\mathrm{final}}} \left( \frac{g}{g} \right),$$

   where **P_final_** represents the butanol concentration at the end of cultivation, **P_0,_** the butanol concentration at the beginning of cultivation, and **S_final_** and **S_0_** the glucose concentrations at the end and beginning of cultivation respectively.

   Butanol production rate was calculated using the following equation:

$$r_{\mathrm{butanol}}=\frac{P_{\mathrm{final}}-P_{0}}{t_{\mathrm{final}}-t_{0}} \left( \frac{g}{L\cdot h} \right),$$

   where **P_final_** represents the butanol concentration at the end of cultivation, **P_0,_** the butanol concentration at the beginning of cultivation, and **t_0_** and **t_final_** cultivation times of the beginning and end of the cultivation respectively.

   Glucose consumption rate was calculated using the following equation:

$$r_{glucose}=\frac{S_{0}-S_{final}}{t_{final}-t_{0}} \left( \frac{g}{L\cdot h} \right),$$

   where **S_final_** is the glucose concentration at the end of cultivation, **S_0_** is the glucose concentration at the beginning of cultivation, and **t_0_** and **t_final_** cultivation times at the beginning and end of the cultivation respectively. [↑](#footnote-ref-2)
3. represents the number of quality checked and mapped reads [↑](#footnote-ref-3)
